# Enhancing mental health resilience in the healthcare workforce during COVID-19: lessons from The North West Offer

**DOI:** 10.1016/j.fhj.2025.100249

**Published:** 2025-04-10

**Authors:** Georgia Lada, Amy L Rathbone, Andy Coley, Jane Cummings, Cecil Kullu, Raj Kumar, Joe Rafferty, Rajan Madhok, Sir Cary L Cooper, C Elise Kleyn

**Affiliations:** a Dermatology Centre, Northern Care Alliance NHS Foundation Trust, Manchester Academic Health Science Centre, Division of Musculoskeletal & Dermatological Sciences, The University of Manchester, Manchester, United Kingdom; b Greater Manchester Mental Health NHS Foundation Trust, Manchester, United Kingdom; c University of East Anglia, School of Health Sciences, Norwich, United Kingdom; d NHS Clinical Leaders Network, United Kingdom; e NHS Gloucestershire, Gloucester, United Kingdom; f Eric Moore Parthership, Warrington, United Kingdom; g Mersey Care NHS Foundation Trust, Liverpool, United Kingdom; h Retired public health doctor, United Kingdom; i Alliance Manchester Business School, The University of Manchester, Manchester, United Kingdom

**Keywords:** Workforce resilience, Clinical leadership, NHS, Healthcare workforce, COVID-19 pandemic

## Abstract

During the COVID-19 pandemic, the NHS Clinical Leaders Network (CLN) developed ‘The North West Offer’ for Enhancing Mental Health Resilience (EMHR) in the north west of England. The EMHR programme aimed to reinforce psychological resilience in the healthcare workforce; that is, enhance the mental, emotional and behavioural adaptation processes of the workforce to pandemic challenges.

The present paper describes the EMHR programme, reviews its evaluation outcomes and proposes a series of recommendations regarding promoting psychological resilience in the healthcare workforce.

Recommendations include establishing reflective support for leaders, promoting knowledge sharing between leaders and organisations, collaborating with a variety of internal and external partners to maximise staff access to psychological services, ensuring uninterrupted access to clinical psychologists for staff use and monitoring of at-risk staff. These recommendations are expected to strengthen mental health workforce resilience for stressors during business as usual as well as future pandemic planning.

## Highlights

• We review the Enhancing Mental Health Resilience-EMHR Project in North West England.
• EMHR aimed to address resilience in the healthcare workforce during COVID-19.
• EMHR included individual leader support and wellbeing interventions for staff.
• A need for staff-dedicated psychologists and empathetic workplace culture was shown.
• Outcomes are expected to inform day-to-day resilience and future pandemic planning.

## Introduction

Following the COVID-19 outbreak, healthcare professionals experienced both professional and personal challenges affecting their mental health, including anxiety, isolation, concern about their health and workplace safety, moral injury and changes in clinical practice.^1^ In response to the challenges, the NHS Clinical Leaders Network (CLN), guided by a multidisciplinary expert Advisory Steering Group led by a senior consultant psychiatrist, reviewed available evidence and produced a report in April 2020, advising how to enhance the mental health resilience of the workforce. This call for action, which identified key priorities for workforce resilience and advocated preparation for the projected impact of COVID-19 on staff,^2^ was responded to positively by healthcare trusts across the north west of England.

As a result of this report, the CLN developed the Enhancing Mental Health Resilience (EMHR) programme to support its membership and the wider NHS workforce. The panel developing the programme consisted of senior GPs, physiotherapists, psychiatrists, psychologists, psychotherapists, paramedics and experienced organisation leaders. Input from a panel of experienced, multidisciplinary professionals was sought to broaden the programme reach across the spectrum of healthcare professionals. The EMHR programme was funded by three regional integrated care boards. It was designed to address the key priorities identified by the call for action, including prioritising the mental health of the workforce in pandemic response plans, coordinating and developing mechanisms to identify staff needs and mitigate long-term effects, collaboration of mental health services with primary care and general hospitals for staff triaging and management, and establishing protected funding. It also addressed the need for compassionate leadership, which involves encouraging compassion and an organisational culture with interpersonal focus through listening, empathy and employee support.^3^

The programme used a three-pronged strategy to enable implementation and evaluation.^4^ Its ultimate aim was to develop a coordinated model to understand and respond in a timely manner to the current mental health crisis in the healthcare workforce, while adding to the limited evidence base regarding reinforcing workforce resilience during future pandemics.

We describe the strategy and review the outcomes from the programme evaluation undertaken collaboratively with the University of Manchester in order to form recommendations for future policy.

## Methodology

Within acute, ambulance, community and mental health, and specialist NHS trusts across the north west of England, clinical commissioning groups and primary care, the CLN engaged stakeholders and then individuals (leaders and staff) into three EMHR initiatives as follows:

First, qualitative semi-structured interviews were conducted with human resources (HR) / workforce directors and clinical psychologists hired for staff use to explore trust alignment to a shared framework, funding, responsibilities and reported mental health issues. Psychologists supported staff via one-to-one sessions, webinars and drop-in sessions in their trusts.

Second, the EMHR introduced individual support for CLN members through digital action learning sets (DALS). DALS included targeted training and monthly online meetings in small groups for leaders, enabling reflective leader skills development with use of reflection frameworks to resolve challenges, supported by trained facilitators. DALS were evaluated using a survey, exploring knowledge acquired and success of actions taken.

Third, the EMHR disseminated the C-19 Shortened Stress Evaluation Tool (C-19 ASSET) questionnaire to staff, which assesses employee job perception, organisational commitment and employee health. C-19 ASSET was adapted from the ASSET questionnaire^5^ by CLN and Robertson Cooper to include 14 COVID-19-specific questions. A tailored wellbeing report was generated upon completion, enabling individuals to identify and prioritise hotspot areas across three categories (psychological health, physical health, positive emotions).

The programme evaluation was based on Donabedian’s healthcare quality model due to its validity, reliability and relevance.^6^ This is a conceptual model suggesting that improved structure results in improved processes and subsequently quality of care.^7^

## Outcomes

The present evaluation reports on activities during June 2020 to April 2021. During this time, the EMHR was offered to 14 NHS trusts across the north west of England, 12 of which participated. Overall, uptake was best at organisational level and less optimal on member level.

Similar themes were identified across the three strategies, although the latter showed different efficacy in addressing those.

During interviews, the organisations reported unanimously that, while concerns in the first pandemic wave focused on transmission, during the second wave, anxiety focused on personal, professional and organisational ability to cope. It was found that executive teams used both internal and external initiatives, including use of mental health first aiders and closed social media platforms. Trusts signed up to the Ask Twice campaign (https://www.time-to-change.org.uk/asktwice), which advocates the continuous monitoring of staff mental health. HR/workforce directors and psychologists evaluated the ongoing provision of mental health initiatives as critical for preventing long-term effects and future crises.

Psychologists working with staff assessed reassurance regarding confidentiality as important for staff. A notable organisational challenge psychologists faced was influencing management to put less pressure on redeployed staff regarding dealing with backlog when returning to usual duties.

Regarding leadership, interviews suggested that leaders/managers were reluctant to disclose personal mental health experiences due to fear of judgment. However, it was recognised that open disclosure would promote understanding between teams. The DALS addressed this issue by encouraging leaders’ reflection and individual initiatives. Feedback from DALS participants suggested that leaders mostly benefited from the opportunity to present problems and use challenge/support from colleagues and experienced fewer benefits regarding their own problem-solving ability. Leaders highlighted successful DALS implementation into their teams, through establishing team time and developing specialty-tailored DALS. However, leaders reported difficulty in aligning schedules for DALS during COVID-19.

Overall, using Donabedian’s model, the EMHR structure and process were assessed as pragmatic and led by the NHS employees’ needs, given the multidisciplinary input in its design and organisational and individual engagement. Regarding EMHR aims and outcomes (improvement of mental health and resilience), the EMHR ensured that resilience in the workforce remained a priority via its direct outreach and multiple initiatives. Regarding the three strategies: (i) Interviews highlighted the importance of the psychologists’ support pathway as a safe disclosure space. However, although the call for action paper reiterated how critical funding is, psychologists were on fixed contracts and counselling sessions were discontinued when contracts ended. (ii) DALS enabled exchange of resilience strategies between organisations and appeared effective in promoting compassionate leadership. (iii) The C-19 ASSET questionnaire, used to identify staff wellbeing needs, provided a snapshot of employee mental health; however, it was not deemed a sufficient tool alone to provide a complete understanding of the staff mental health needs.

## Conclusion and next steps

Several aspects of the EMHR programme were effective in addressing mental health resilience in the healthcare workforce during COVID-19. These can be used to inform ongoing efforts to promote resilience in non-pandemic times and plan for future crises. Although the healthcare workforce is renowned for its resilience in the face of adversity, this should not mean that it should be relied upon for continuation of care, since this may further increase burnout. Growing evidence suggests that resilience can be reinforced through targeted interventions.^8^ Building resilience can help reduce the negative effects of stressors and prevent mental disorders, notably depression.^9^

Lessons learned from previous pandemics suggest that psychological protective factors are being ‘mentally healthy’ and having strong support systems, pre- and post-crisis.^10^ The EMHR programme provided support at the individual worker, clinical leader/manager and organisational level. Outcomes supported the need for ongoing structured, region- or nationwide efforts to promote psychological resilience in the workforce. Based on these outcomes, we propose seven recommendations to improve resilience (Table 1).^4^

**Table 1:** Recommendations for enhancing mental health resilience in the healthcare workforce

EMHR programme recommendations
• Focus on reflective support for leaders is important to enable individual resilience, as well as fostering of a favourable environment for their teams/organisations. • Action learning sets delivered remotely for clinical leaders/managers may be helpful to enable knowledge sharing of best practice noun for the benefit of healthcare professionals. • Organisations would benefit by working in collaboration with a variety of partners to maximise access to a spectrum of mental health services for their staff members. • Active engagement with the Ask Twice campaign is recommended to provide staff with the space to discuss mental health conditions. • NHS staff would benefit from a commitment to have uninterrupted access to clinical psychologists. • It is important that there is vigilance within organisations to flag staff at risk of mental health conditions as well as being mindful of avoiding marginalisation of individuals. • Organisations should be aware that pandemics expose staff to high stress environments and therefore mental conditions may arise or be exacerbated throughout the pandemic periods. Early action to ameliorate the impact is critical to mental health resilience.

Note: Recommendations are adapted from our initial full EMHR evaluation report ‘Enhancing Mental Health Resilience Final Report 2021″ (p. 27), as published in the NHS CLN website.^4^

At an organisational level, continued mental health service provision was assessed as important by psychologists and organisations to prevent future crises. This should include both access to staff psychologists and initiatives to support, promote confidence and enhance job autonomy for individual staff. Staff risk assessments combined with a compassionate leadership approach^11^ and ongoing support offer should be used as early intervention to identify physical and psychological health problems. Team-building exercises, safe spaces for staff and closed social media groups/platforms were also valued and could be promoted within trusts.

At the individual level, the C-19 ASSET questionnaire is an example of a tool providing personalised feedback, which may be useful to employees. Of note, pre- and post-pandemic, the original ASSET is used to help organisations to identify areas where they can improve employee wellbeing. It is critical that organisations continue to focus on employee wellbeing, particularly given the post-pandemic pressures.

At leadership level, targeting leaders to promote reflective, compassionate leadership emerged as a key strategy to improve leaders’ skills and wellbeing, and helps establish a culture of open discourse within organisations. DALS continuation outside of the pandemic context would encourage multi-partner, collegial support across acute, community and primary services.

Limitations arising from the EMHR programme should also be addressed to improve ongoing strategy implementation. This includes boosting funding to enable permanent employment of staff psychologists and establishing protected time for leaders to attend DALS.

Overall, we believe that recommendations arising from the EMHR programme continue to be relevant in the current landscape. These should inform strategic planning in future initiatives and are important both in view of future crises as well as during business as usual.

## Ethics approval and consent to participate

Not applicable.

## Funding

The programme evaluation and case study did not receive any specific grant from funding agencies in the public, commercial or not-for-profit sectors. The EMHR programme was funded by three regional integrated care boards.

## Data availability statement

The data that support the findings of this study are available from the corresponding author upon reasonable request.

## CRediT authorship contribution statement

**Georgia Lada:** Writing – review & editing, Writing – original draft, Visualization, Software, Methodology, Conceptualization. **Amy L Rathbone:** Writing – review & editing, Writing – original draft, Visualization, Software, Methodology, Conceptualization. **Andy Coley:** Writing – review & editing, Resources, Methodology, Data curation, Conceptualization. **Jane Cummings:** Writing – review & editing, Resources, Methodology, Data curation, Conceptualization. **Cecil Kullu:** Writing – review & editing, Resources, Methodology, Data curation, Conceptualization. **Raj Kumar:** Writing – review & editing, Resources, Methodology, Data curation, Conceptualization. **Joe Rafferty:** Writing – review & editing, Resources, Methodology, Data curation, Conceptualization. **Rajan Madhok:** Writing – review & editing, Resources, Methodology, Data curation, Conceptualization. **Sir Cary L Cooper:** Writing – review & editing, Resources, Methodology, Data curation, Conceptualization. **C Elise Kleyn:** Writing – review & editing, Supervision, Resources, Methodology, Data curation, Conceptualization.

## Declaration of competing interest

The authors declare the following financial interests/personal relationships which may be considered as potential competing interests: RK is the chair of NHS Clinical Leaders Network and the deputy chief medical information officer for NHS England. JR is the chief executive officer of Mersey Care NHS Foundation Trust. AC is the chief clinical officer of the NHS Clinical Leaders Network. JC is the chair of the Royal College of Nursing (RCN) Foundation and non-executive director for NHS Gloustershire. CLC is co-founder of Robertson Cooper, president of the Institute of Welfare and chair of the National Forum for Health & Wellbeing at Work. GL has received speaker honoraria from Janssen, Lilly, Leo and Novartis. CEK conflicts of interest are (including consulting fees, research or institutional support, and educational grants): Almirall, Amgen, Celgene, Eli Lilly and Company, Janssen Pharmaceuticals, La Roche-Posay, LEO Pharma, Novartis, Pfizer and UCB. CEK is also a visiting professor at the University of Bolton. CK, RM and ALR declare that they have no known competing financial interests or personal relationships that could have appeared to influence the work reported in this paper.
